# An Evaluation Model for Brain Ischemia Protection in Mice by Low-Intensity Pulsed Ultrasound Stimulation Based on Functional Cortico-Muscular Coupling

**DOI:** 10.3390/bioengineering12050541

**Published:** 2025-05-17

**Authors:** Ziqiang Jin, Xiaoling Chen, Zechuan Du, Yi Yuan, Xiaoli Li, Ping Xie

**Affiliations:** 1 Institute of Electric Engineering, Yanshan University, Qinhuangdao 066004, China; xlchen@ysu.edu.cn (X.C.); duzechuan@stumail.ysu.edu.cn (Z.D.); yuanyi513@163.com (Y.Y.); 2 Key Laboratory of Intelligent Rehabilitation and Neuromodulation of Hebei Province, Yanshan University, Qinhuangdao 066004, China; 3 National Key Laboratory of Cognitive Neuroscience and Learning, Beijing Normal University, Beijing 100875, China; xiaoli@bnu.edu.cn

**Keywords:** ischemic stroke, low-intensity pulsed ultrasound stimulation, bilateral carotid artery occlusion model, functional cortico-muscular coupling, evaluation model

## Abstract

(1) Background: Ischemic stroke is a major global public-health concern with complex pathogenesis. Current treatment strategies face challenges. Low-intensity pulsed ultrasound stimulation (LIPUS), a non-invasive neuromodulation technology, shows promise in treating ischemic stroke, yet its underlying mechanisms lack in-depth investigation, especially in quantitative efficacy evaluation. (2) Methods: This study aimed to develop a neuromuscular functional coupling-based dynamic time warping (DTW) model to evaluate LIPUS’s neuroprotective effects in a mouse model of ischemic stroke. A bilateral carotid artery occlusion (BCAO) model in mice was established, and LIPUS treatment was given. Time- and frequency-domain analyses of local field potentials (LFPs) and electromyography (EMG) were conducted, and outcomes were quantified using a percentage-based scoring system. (3) Results: The BCAO+LIPUS group scored significantly higher than the BCAO group. (4) Conclusions: This study demonstrated that LIPUS is neuroprotective in BCAO mice and that the DTW-100 assessment evaluation model can quantify the neuroprotective effects of LIPUS.

## 1. Introduction

Ischemic stroke, a category of cerebrovascular disorders characterized by high rates of morbidity, disability, and mortality, has been widely recognized as a significant global public health concern [1]. The pathogenesis of ischemic stroke involves complex, multidimensional interactions, including—but not limited to—pathophysiological processes such as neuronal apoptosis, neuroinflammatory cascades, and disrupted energy metabolism, which often result in irreversible motor dysfunction and severely compromise the quality of life [2].

Current clinical intervention strategies for ischemic stroke primarily include intravenous thrombolytic therapy [3,4,5], endovascular mechanical thrombectomy [6,7,8], and the administration of neuroprotective agents [9,10,11], among others. However, the clinical application of these therapies—particularly the invasive approaches—remains significantly challenged by factors such as a narrow therapeutic time window, the risk of secondary hemorrhage, and limited efficacy in promoting functional recovery. In recent years, low-intensity pulsed ultrasound stimulation (LIPUS), an emerging non-invasive neuromodulation technology in the fields of rehabilitation and physical therapy [12], has demonstrated promising potential in the treatment of ischemic stroke, owing to its non-invasive nature, safety profile, and ease of application. Studies have shown that LIPUS exerts neuroprotective effects by enhancing cerebral blood circulation, promoting neural regeneration, and attenuating inflammatory responses [12,13,14]. J. Chen et al. reported that post-stroke inflammation plays a critical role in cerebral ischemic injury and that its modulation can mitigate neurological damage [15]. Similarly, Guo et al. demonstrated that LIPUS treatment can attenuate cerebral ischemic injury by enhancing cerebral blood flow and reducing neutrophil infiltration [16]. C.-M. Chen et al. and Wu et al. demonstrated that LIPUS pretreatment significantly attenuates cerebral ischemic injury induced by middle cerebral artery occlusion (MCAO), improves balance perception and motor function, and further substantiates the neuroprotective effects of LIPUS through microglial studies [13,14]. However, despite the growing body of evidence supporting the therapeutic potential and beneficial effects of LIPUS, in-depth investigations into its underlying mechanisms remain limited—particularly concerning quantitative methods for evaluating its efficacy in treating cerebral ischemia. Most current studies rely on behavioral observations or pathological assessments to evaluate treatment outcomes [14,17] and lack a quantitative evaluation framework for treatment efficacy grounded in neuromuscular functional coupling. Arya and Pandian noted that neuromuscular functional coupling refers to the close interaction between the nervous and muscular systems, which is essential for maintaining standard motor and physiological functions [11]. Clark et al. found that following cerebral ischemia, partial central nervous system dysfunction leads to a reduction in neuromuscular functional coupling, prompting the body to adopt compensatory movement patterns [18]. This disruption results in decreased joint mobility and reduced muscle utilization, culminating in motor dysfunction [19,20,21,22]. Therefore, the therapeutic efficacy of LIPUS on cerebral ischemia, as assessed through neuromuscular functional coupling and supported by synchronized signal acquisition, is of substantial scientific significance.

Since neuromuscular functional coupling analysis requires the simultaneous acquisition of EEG and EMG signals, dynamic time warping (DTW) algorithms—initially developed for speech recognition—have been widely applied across various fields, including speech recognition [23,24,25,26], bioinformatics [27,28,29], financial analysis [30,31,32], and other fields. The DTW algorithm minimizes the cumulative distance between two sequences by constructing a dynamic alignment path to identify the optimal temporal warping function, thereby overcoming the limitations of traditional Euclidean distance, which assumes rigid alignment along the time axis [33]. In recent years, with the growing prominence of multimodal data processing, the robustness of DTW in cross-domain sequence alignment has been increasingly recognized, establishing it as a vital tool for time-series similarity measurement [34,35,36].

Building upon our previous work [37], the present study aimed to develop a model based on neuromuscular functional coupling for evaluating the neuroprotective effects of low-intensity pulsed ultrasound stimulation in a mouse model of ischemic stroke. By establishing a bilateral carotid artery occlusion (BCAO) model in mice and administering LIPUS treatment, the effects of LIPUS on neuromuscular functional coupling following ischemic stroke were evaluated through time- and frequency-domain analyses of local field potentials (LFPs) and electromyography (EMG), with outcomes quantified using a percentage-based scoring system. The findings of this study are expected to offer new insights into the quantitative evaluation of LIPUS, supporting its future clinical applications by assessing its rehabilitative and neuroprotective effects in subjects before and after stimulation.

## 2. Materials and Methods

### 2.1. Animal Anesthesia and Surgery

**Figure 1:**
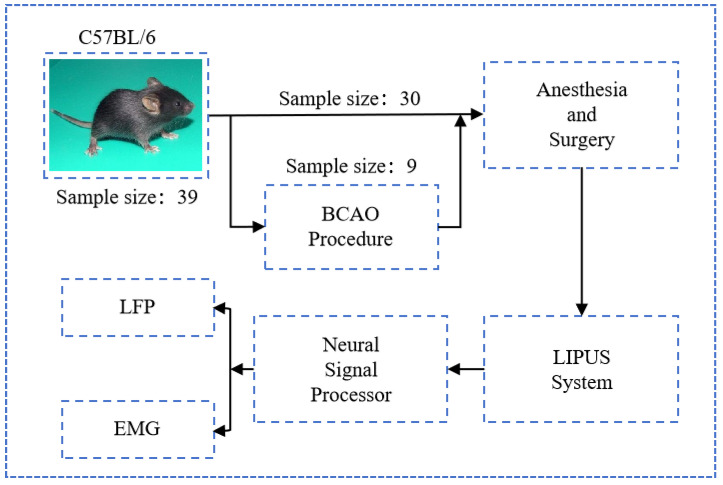
Experimental process diagram.

As depicted in Figure 1, a total of 39 male C57BL/6 mice (all male, body weights 20–25 g, Beijing Vital River Laboratory Animal Technology Co., Ltd., Beijing, China) were utilized in the experimental procedures; nine of them underwent BCAO surgical modeling. All experimental protocols were conducted in strict compliance with the guidelines established by the Animal Ethics and Administrative Council of Yanshan University and Hebei Province, People’s Republic of China. Surgical anesthesia was administered through intraperitoneal injection of sodium pentobarbital (3%, 5 mg/100 g body weight). Following anesthesia induction, the animals were securely positioned in a stereotaxic apparatus (model: 6800268030, RWD Co., Shenzhen, China). The cranial region was prepared by shaving the scalp hair and disinfecting the area with normal saline. A midline incision was made along the sagittal suture, followed by meticulous removal of connective tissue and periosteum to expose the skull surface. Utilizing stereotaxic coordinates derived from the mouse brain atlas, precise identification of the motor cortex was achieved. Upon completion of experimental procedures, euthanasia was performed through administration of an anesthetic overdose by institutional animal care protocols.

### 2.2. BCAO Procedure

The murine disease model was generated through BCAO, following established protocols. In summary, mice were subjected to deep anesthesia induced by 2.5% isoflurane delivered via a vaporizer system. The anterior cervical region was sterilized with 75% ethanol prior to creating a midline incision. The right common carotid artery was meticulously dissected and encircled with a micro-coil (0.18 mm internal diameter). An identical surgical intervention was subsequently conducted on the left common carotid artery. The incision was then closed with sutures. Throughout the procedure, the core body temperature of the mice was regulated at 37 ± 0.5 °C using a homoeothermic heating system (Harvard Homoeothermic Blanket Control Unit, Harvard Apparatus, Holliston, MA, USA). To mitigate the risk of postoperative infection, a penicillin sodium solution was applied to the surgical wounds.

### 2.3. Low-Intensity Pulsed Ultrasound Stimulation System

**Figure 2:**
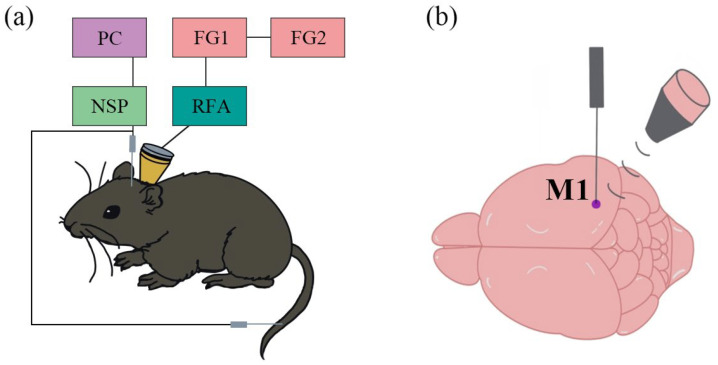
(**a**) The experimental system. (**b**) The stimulus location and electrode insertion position. (FG: function generator, RFA: RF amplifier, NSP: neural signal processor, PC: personal computer).

Two signal generators (AFG3022C, Tektronix, Beaverton, OR, USA) were employed to generate continuous modulated sine wave and square wave signals, which were combined to form pulsed waveforms. The modulated signals were amplified using a power amplifier (E&I 240L, ENI Inc., New York, NY, USA) and subsequently delivered through a focused ultrasound probe (V301-SU, Olympus, Center Valley, PA, USA; focal point diameter: 25.4 mm, curvature radius: 40 mm). A conical collimator was positioned between the ultrasound probe and the mouse’s head, fabricated from 3D-printed resin material with a 4 mm diameter and filled with ultrasound coupling fluid. This setup ensured the effective transmission of ultrasound waves through the scalp and skull, targeting the primary motor cortex (M1) as guided by the mouse brain atlas. The ultrasound parameters were configured as follows: fundamental frequency = 500 kHz, pulse repetition frequency = 1 kHz, duty cycle = 50%, stimulation duration = 300 ms, and inter-trial interval = 4 s. The spatial peak pulse average intensity (Isppa) at the tail motor cortex was measured using a hydrophone and determined to be 0.95 W/cm^2^. Each sample underwent 30 cycles of testing, and the entire experimental system is shown in Figure 2a.

### 2.4. Data Acquisition

A micro tungsten electrode (WE50030.1B10, MicroProbe, Gaithersburg, MD, USA) was stereotaxically implanted into the primary motor cortex (M1) to acquire LFP. Simultaneously, EMG signals were recorded using a modified single-channel microelectrode (WE50030.1B10, MicroProbe, USA) inserted into the tail muscles. The signals from these electrodes were transmitted through a dual-channel preamplifier (63386, A-M Systems Inc., Carlsborg, DC, USA) to a neural signal acquisition system (Cerebus, Blackrock Microsystems, Salt Lake City, UT, USA) for subsequent storage and analysis. The sampling rates were set at 30 kHz for the low-intensity pulsed ultrasound (LIPUS) signals and 2 kHz for both LFP and EMG recordings. A schematic representation of the experimental setup is provided in Figure 2b.

### 2.5. Data Processing and Statistics

**Figure 3:**
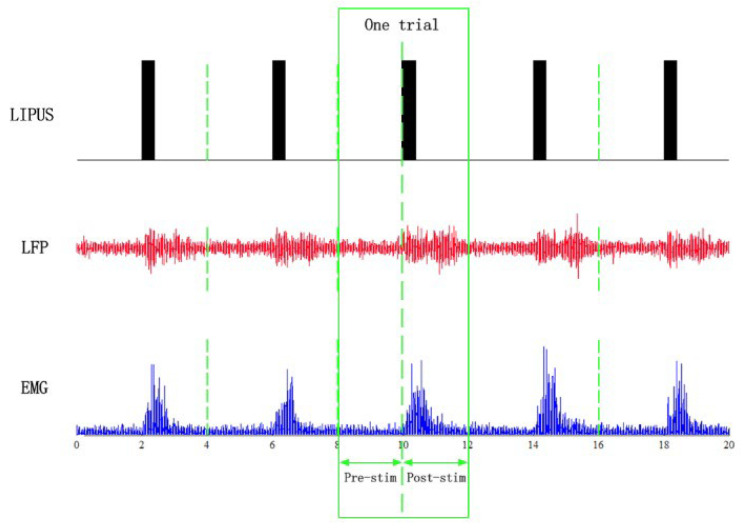
LIPUS and the LFP and EMG signals after preprocessing.

To avoid IF interference in the original LFP and EMG recordings, a trap filter was used to remove 50 Hz IF signals and an adaptive high-pass filter was used to remove baseline drift. The data period was 4 s. A 0.5–200 Hz bandpass filter was used for the LFP and a 10–200 Hz bandpass filter was used for the EMG. signals before and after LIPUS were intercepted. Data were collected from 30 trials per group and averaged by superposition in Figure 3. The normal group of 30 samples was averaged to ensure stability of the baseline reference as a standard for DTW calculations. The BCAO samples were a total of nine, each of which was surgically modeled. Both the normal group and BCAO group were subjected to resting for 4 h, waiting for the surgical anesthesia to wear off to avoid anesthesia interference. Statistical analysis was performed using *t*-tests.

### 2.6. Dynamic Time Warping Analysis

**Figure 4:**
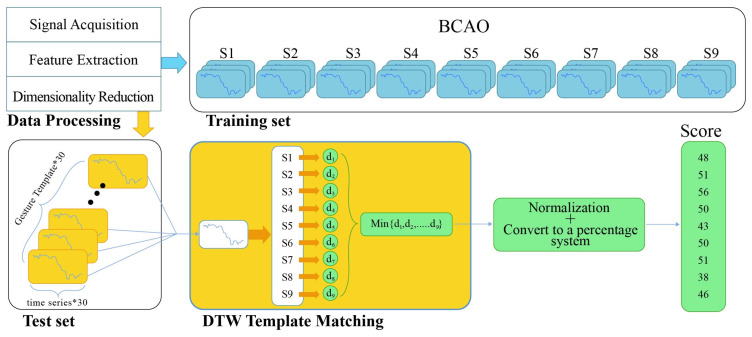
Improved DTW-100 flowchart based on the DTW algorithm.

As shown in Figure 4, the mean values of LFP and EMG collected from 30 normal mice were used as the reference level for the DTW-100 algorithm. The DTW distance was calculated for LFP and EMG of nine BCAO samples, and the distance was converted into a percentage score through normalization and percentage scale changes.

The interplay between LFP and EMG signals indicates the functional connectivity between the motor cortex and effector muscles. To quantitatively assess the impact of LIPUS on the rehabilitation of BCAO mice, the dynamic time warping (DTW) algorithm was employed. The DTW analysis was conducted using two reference datasets: (1) LFP and EMG recordings from normal mice in a resting state, and (2) LFP and EMG signals from BCAO mice under LIPUS stimulation. These reference datasets were compared against the LFP and EMG signals obtained from BCAO mice subjected to LIPUS. The resulting DTW values were normalized and converted into percentage scores to derive the respective DTW scores, providing a quantitative measure of the rehabilitation effect induced by LIPUS.

Define the LFP and EMG signals as two time sequences X = (x_1_, x_2_, x_3_,⋯, x_n_) and Y = (y_1_, y_2_, y_3_,⋯, y_n_), where n is the length of the two sequences. The goal of DTW is to find an alignment path P = (p_1_, p_2_,⋯, p_k_),where each p_i_ = (a_i_, b_i_) denotes the corresponding point between X and Y. The DTW is performed by using the following method.

First, the distance matrix D between two sequences is calculated, with D(i, j) representing the Euclidean distance between x_i_ and y_i_.

(1) $$D\left(i,\;j\right)={\left|x_{i}-y_{i}\right|}^{2}$$

Next, the cumulative distance matrix C is computed, where C(i, j) denotes the minimum cumulative distance from (11) to (i, j). The cumulative distance matrix is computed as:

(2) $$C\left(i,j\right)=D\left(i,j\right)+min\left\{C\left(i-1,j\right),C\left(i,j-1\right),C\left(i-1,j-1\right)\right\}$$

The final DTW distance is the last element of the cumulative distance matrix:

(3) $$DTW\left(x,y\right)=C\left(n,m\right)$$

where C(n,m) is the last element of the cumulative distance matrix and represents the minimum cumulative distance from the start to the end of the sequence.

Since the value of DTW distance is affected by the length and amplitude of X earlier, the DTW distance is normalized:

(4) $$DTW_{norm}\left(X,Y\right)=DTW\left(X,Y\right)/2n$$

where n is the length of X, Y.

DTW distances were converted to DTW percentiles based on normalization.

(5) $$DTW-100{\left(X,Y\right)}_{LFP}=100\times \left(1-A\times DTW_{norm}\left(X,Y\right)\right)$$

(6) $$DTW-100{\left(X,Y\right)}_{EMG}=100\times \left(1-A\times DTW_{norm}\left(X,Y\right)\right)$$

where DTW-100 is the percentage scoring function of the DTW algorithm and A is the adjustable coefficient.

(7) $$DTW-100\left(X,Y\right)=0.5\times DTW-100_{LFP}\left(X,Y\right)+0.5\times DTW-100_{EMG}\left(X,Y\right)$$

where the total DTW-100 score is 0.5 multiplied by the sum of the LFP and EMG scores and 0.5 is an individually defined scaling parameter.

## 3. Results

### 3.1. Preprocessed Data Graphs of Normal and BCAO Mice Before and After LIPUS

**Figure 5:**
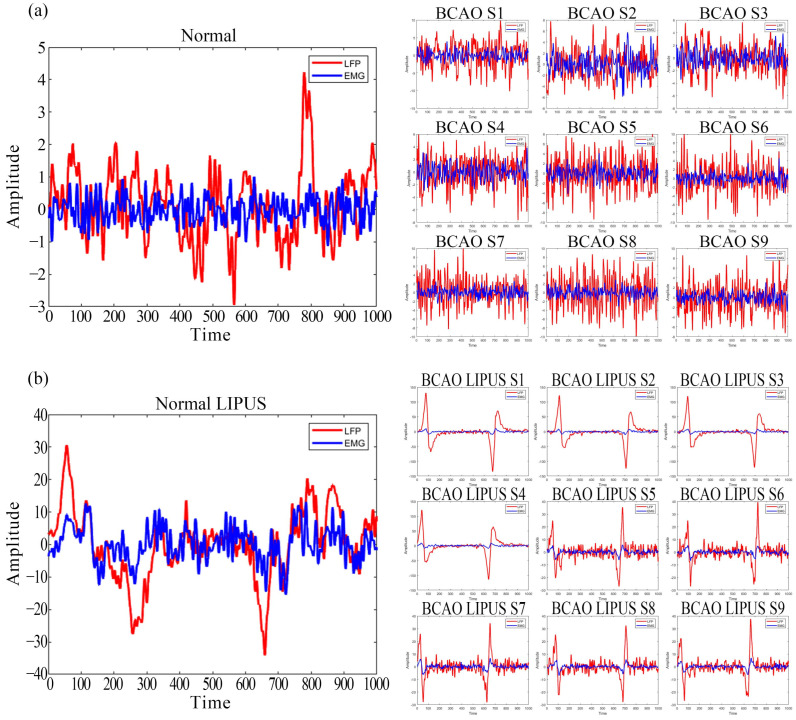
(**a**) Preprocessed data graph of mean normal and BCAO mice before LIPUS. (**b**) Preprocessed data graph of normal and BCAO mice after LIPUS.

As depicted in Figure 5a, averaged LFP and EMG signals from 30 trials in normal mice were compared with pre-stimulation data from nine BCAO models. The results demonstrate that the LFP signal in normal mice was slightly stronger than the corresponding EMG. In contrast, BCAO mice exhibited a significantly higher LFP amplitude, with peak magnitudes nearly three times greater than EMG. Additionally, LFP and EMG signal variability were markedly increased in BCAO mice compared to the normal group.

As depicted in Figure 5b, post-stimulation LFP and EMG signals in normal mice showed a marginal LFP dominance over EMG. However, in BCAO mice, the LFP amplitude far exceeded EMG levels, reaching a maximum magnitude approximately 100-fold higher. Signal variability remained significantly elevated in the BCAO group.

### 3.2. BCAO Group DTW-100 Scores Before and After LIPUS

**Figure 6:**
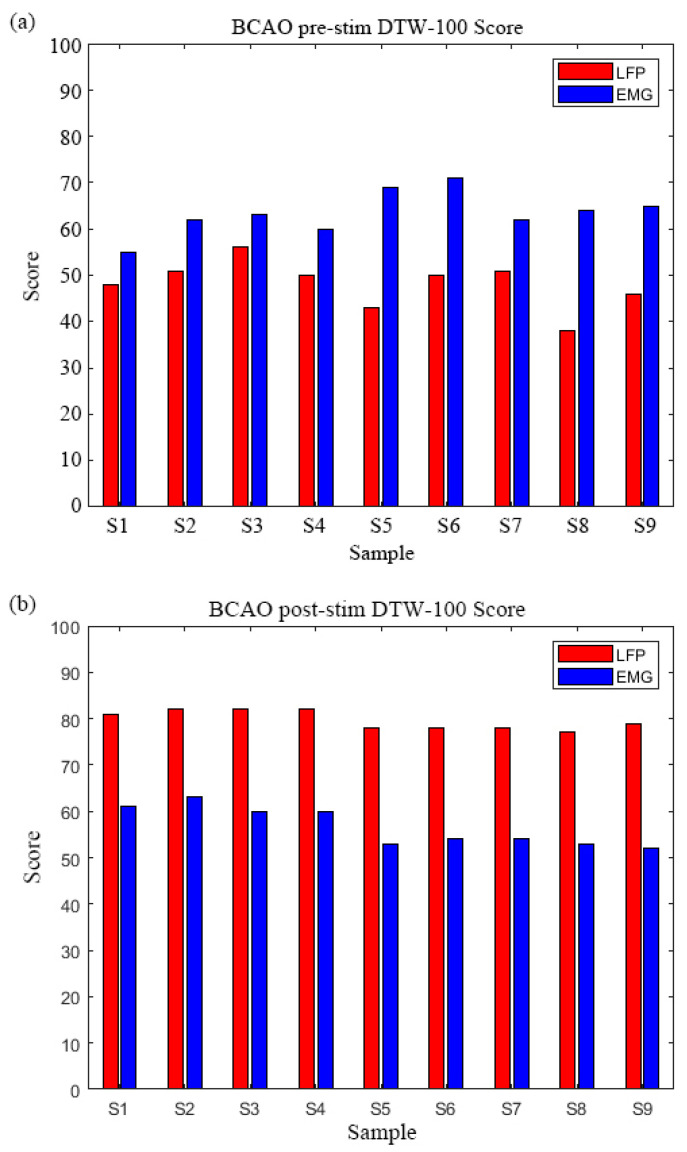
(**a**) BCAO mice DTW-100 scores before LIPUS. (**b**) BCAO mice DTW-100 scores after LIPUS.

As depicted in Figure 6a, comparative DTW-100 analysis of the nine BCAO samples revealed consistent EMG dominance, with EMG scores significantly exceeding corresponding LFP values across all samples, LFP DTW-100 range: 55–71, EMG DTW-100 range: 38–56. The most pronounced disparity was observed in Samples 5 and 6. Group-level analysis showed mean EMG scores (62.3 ± 5.2) were 24.7% higher than LFP scores (48.1 ± 4.9).

As depicted in Figure 6b, the inverse pattern emerged post-stimulation, with LFP scores demonstrating marked elevation over EMG, LFP DTW-100 range: 77–82, EMG DTW-100 range: 52–63. Samples 2–4 exhibited identical peak LFP scores while maintaining stable EMG values. The group mean LFP score (79.7 ± 1.8) surpassed EMG values (56.7 ± 4.3) by 40.6%, with maximal contrast in Sample 9.The DTW-100 score of each sample is shown in Table A1.

### 3.3. DTW-100 Scores

**Figure 7:**
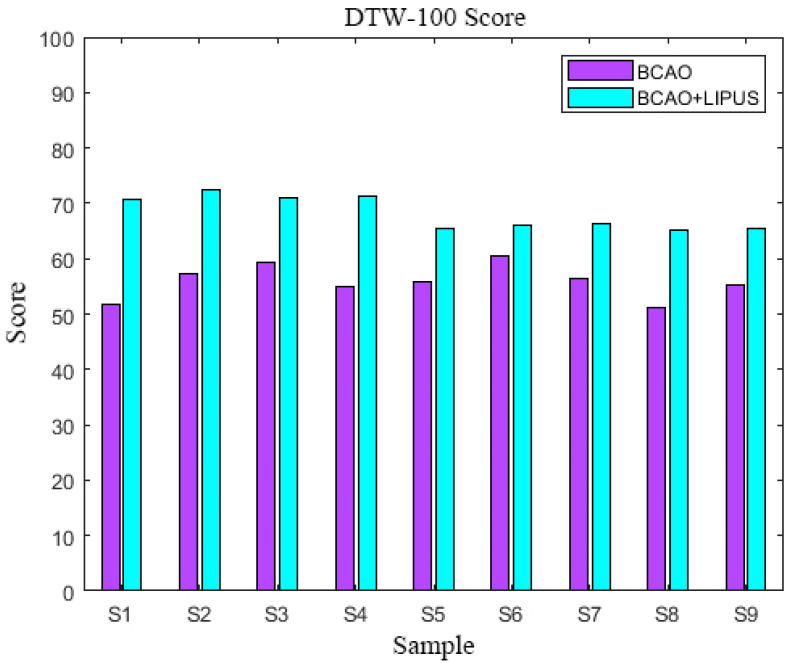
DTW-100 scores.

As depicted in Figure 7, the composite DTW-100 scores, derived from the weighted summation of pre-stimulation LFP and EMG values, revealed significant enhancement following LIPUS intervention. Comparative analysis demonstrated a consistent elevation in DTW scores across all BCAO samples after LIPUS treatment. The mean increase in DTW-100 scores was 14.4 ± 3.6.The DTW-100 score of each sample is shown in Table A1.

### 3.4. Preprocessed Frequency-Domain Data Graphs of Normal and BCAO Mice Before and After LIPUS

**Figure 8:**
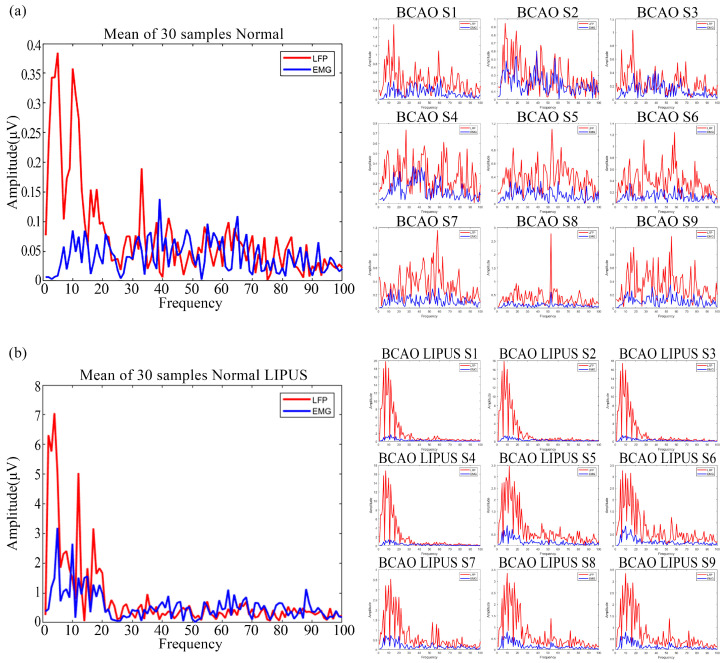
(**a**) Preprocessed frequency-domain data graph of normal and BCAO mice before LIPUS. (**b**) Preprocessed frequency-domain data graph of normal and BCAO mice after LIPUS.

As depicted in Figure 8a, the frequency-domain signals of averaged LFP and EMG from 30 trials in normal mice were compared with pre-stimulation frequency-domain signals from nine BCAO models. The results show that the overall energy of the LFP frequency-domain signals of normal mice is slightly larger than that of the corresponding EMG frequency-domain signals. The energy was concentrated in the 40 Hz frequency range. In contrast, the LFP amplitude of the nine BCAO mice significantly exceeded that of the EMG, with the maximum LFP amplitude nearly four times that of the EMG. The energy was not concentrated in the lower frequency band. In addition, the variability of LFP and EMG signals was significantly higher in the nine BCAO mice compared with the normal group.

As depicted in Figure 8b, the post-stimulation frequency domain of LFP and EMG signals in normal mice showed a marginal LFP dominance over EMG. However, in BCAO mice, the LFP amplitude far exceeded EMG levels, reaching a maximum magnitude approximately 10-fold higher. The energy is concentrated in the frequency range of 40 Hz, and the frequency-domain energy of EMG is significantly reduced. In addition, compared with the normal group, the variability of LFP and EMG signals in nine BCAO mice was considerably lower.

### 3.5. BCAO Group Frequency-Domain DTW-100 Scores Before and After LIPUS

**Figure 9:**
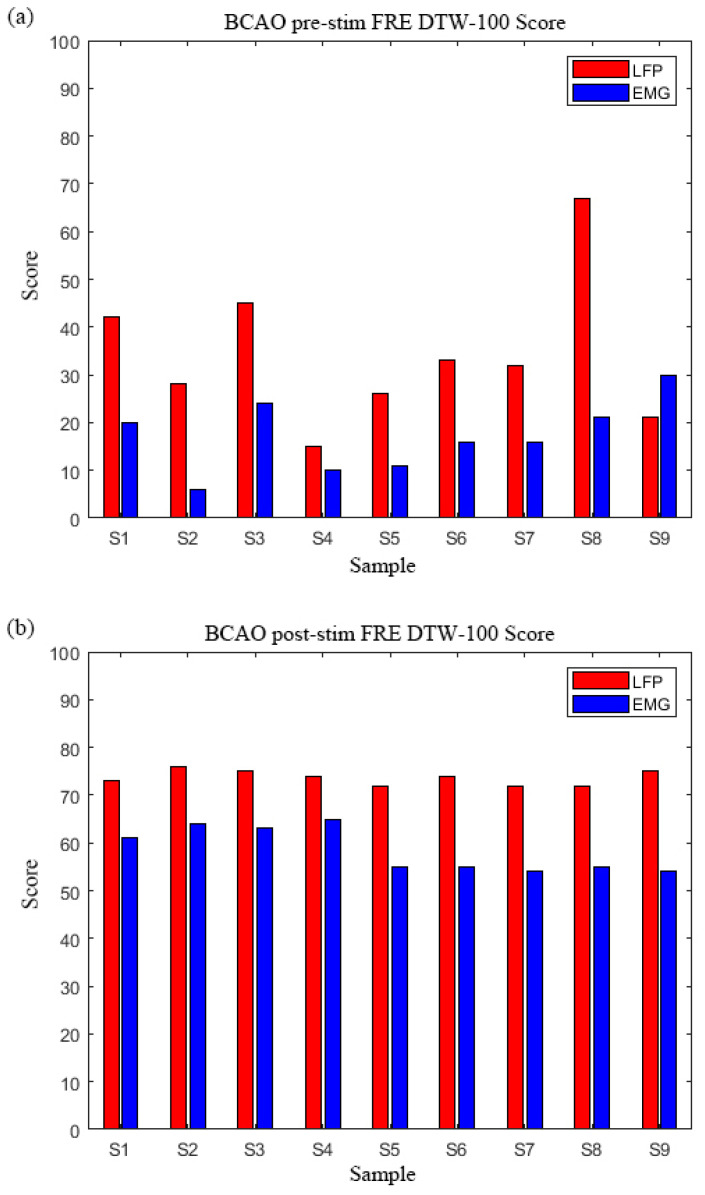
(**a**) BCAO group FRE DTW-100 scores before LIPUS. (**b**) BCAO group FRE DTW-100 scores after LIPUS.

Figure 9a demonstrates a consistent pattern of higher Frequency -(FRE) DTW-100 scores in LFP compared to EMG across all nine BCAO samples. The LFP DTW-100 scores range: 15–67 (mean: 34.3 ± 16.2), while EMG scores were substantially lower, EMG DTW-100 scores range: 6–30. (mean: 17.1 ± 8.1). Sample 8 exhibited the most pronounced difference, while Sample 9 showed the smallest margin.

This trend was further amplified in Figure 9b, where LFP DTW-100 scores consistently exceeded EMG values, with LFP DTW-100 scores ranging from 72–76 (mean: 74.0 ± 1.3), and LFP DTW-100 scores ranging from 54–65 (mean: 58.7 ± 4.4). The most stable LFP performance was observed in Samples 2–6, while EMG scores showed greater variability across samples. Particularly noteworthy was Sample 5, demonstrating a 34.5% higher LFP DTW-100 score than its EMG DTW-100 score.The DTW-100 score of each sample is shown in Table A1.

### 3.6. FRE DTW-100 Scores

**Figure 10:**
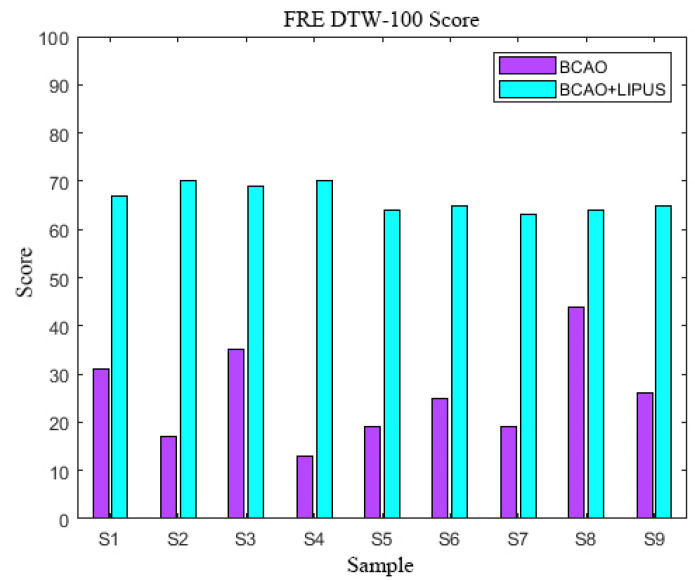
FRE DTW-100 scores.

As depicted in Figure 10, the composite FRE DTW-100 scores, derived from the weighted summation of pre-stimulation LFP and EMG FRE DTW-100 scores, revealed significant enhancement following LIPUS intervention. The pre-stimulation range is 13–44 (mean: 26.0 ± 9.8), while post-stimulation FRE DTW-100 scores range from 63–70 (mean: 66.4 ± 2.7), representing a 2.6 times mean improvement.

### 3.7. Statistical Analysis

**Figure 11:**
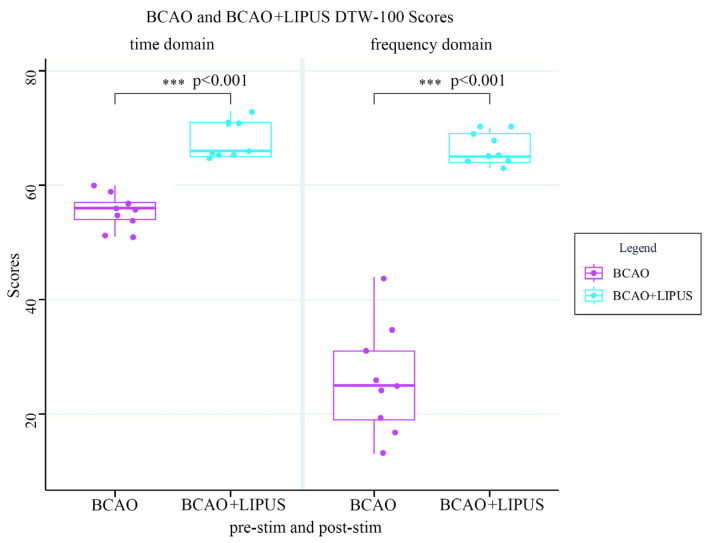
BCAO group and BCAO+LIPUS group DTW-100 scores. *** *p* < 0.001.

In Figure 11, the DTW-100 scores of the BCAO group and the BCAO+LIPUS group are shown in the time and frequency domains. In the time domain, the score of the BCAO group was significantly lower than that of the BCAO+LIPUS group (*p* < 0.001), indicating that LIPUS significantly impacts the time-domain DTW-100 score of BCAO mice. In the frequency domain, the score of the BCAO group was considerably lower than that of the BCAO+LIPUS group (*p* < 0.001), indicating that LIPUS significantly impacted the frequency-domain DTW-100 score of BCAO mice. The BCAO model score in the frequency domain is lower than in the time domain, indicating that the LFP and EMG effects of BCAO surgery on mice are mainly concentrated in the frequency domain. The time-domain and frequency-domain results after stimulation are similar, proving that the neuroprotective effect of LIPUS is superior at the frequency-domain level compared to the time-domain level.

## 4. Discussion

As a noninvasive neuromodulation technique, LIPUS holds significant scientific value in neurological research, mainly through the comparative analysis of pre- and post-stimulation data [38]. This study employed a DTW-based burst similarity computation method, effectively accommodating time-series data of varying lengths and temporal dynamics. This approach enables nonlinear alignment of signal features across time scales and is particularly well-suited for capturing dynamic changes in neural signal analysis. We evaluated the extent of neuromuscular coupling recovery in LIPUS-treated BCAO model mice by conducting behavioral assessments and electrophysiological recordings before and after stimulation [39,40,41]. Given DTW’s capacity to quantify subtle temporal discrepancies, we propose that the DTW-based scoring method offers a more precise and sensitive evaluation of neuromuscular coupling dynamics.

The study’s results indicated that, in the time-domain analysis, the mean LFP values across 30 standard mouse samples were higher than the corresponding mean EMG values. Additionally, segments with markedly elevated LFP energy were observed, possibly associated with spontaneous event-related potentials (ERPs) in mice [42]. In the subject group, comprising nine BCAO model mice, LFP values were significantly higher than EMG values, with the LFP–EMG difference exceeding that observed in the standard group. Furthermore, the LFP values in the BCAO models were markedly elevated compared to those of the normal mice. We hypothesize that the observed transient elevation in LFP among BCAO model mice may reflect heightened neural activity during the acute phase of cerebral ischemic symptoms. This phenomenon differed markedly from observations made after 24 h and may be attributed to variations in neuronal firing patterns during the dying and death phases of brain cells [43]. The higher EMG scores relative to LFP in the pre-stimulation time-domain analysis suggest a disruption in motor control loops—indicating that the BCAO model predominantly impairs central neural pathways while exerting minimal inhibitory effects on effector muscles and peripheral neural circuits.

Post-stimulation LFP signals were slightly higher in the standard group than EMG signals. In contrast, the nine BCAO model mice exhibited a significant elevation in LFP signals following stimulation, characterized by a bimodal fluctuation pattern, consistent with findings from previous studies [37]. Given that the EMG signals in the BCAO models were lower than those observed in the standard group, we hypothesize that effector muscle activity may be suppressed at the cortical level, preventing the signals from fully propagating through the complete motor control loop. Post-stimulation DTW-100 scores of LFP signals were significantly higher than those of EMG in all nine BCAO model samples. Moreover, LFP scores showed a marked increase compared to pre-stimulation values, whereas EMG scores demonstrated a significant decline. Weighted summation analysis of DTW-100 scores for LFP and EMG before and after stimulation revealed that LIPUS significantly enhanced the overall DTW-100 scores in the BCAO model, providing objective evidence of its neuroprotective effects.

Frequency-domain analysis revealed that, during the pre-stimulation period in the 30 standard mouse samples, LFP signals exhibited significantly higher power than EMG signals within the low-frequency band, with energy predominantly concentrated in this range. In nine BCAO model mice, LFP and EMG signals in the frequency domain were elevated compared to the standard group. Unlike the expected condition, the signal energy was no longer confined to the low-frequency band but exhibited a broader distribution across the frequency spectrum. In the pre-stimulation frequency-domain analysis, DTW-100 scores of LFP were generally higher than those of EMG across the samples, except Sample 9. The relative dispersion observed in the LFP energy distribution may be attributed to inter-individual differences in brain structure and also suggests that frequency-domain LFP signals may encode more complex or informative neural dynamics, warranting further in-depth investigation. Post-stimulation DTW-100 scores in the frequency domain demonstrated that LFP signals were significantly higher than EMG signals, indicating that LIPUS exerted a pronounced enhancing effect on LFP activity in BCAO model mice. In contrast, frequency-domain EMG signals exhibited a slight inhibitory response. These findings suggest the presence of adaptive regulatory mechanisms within the nervous system [44,45], and indirectly support the concept of neuroplasticity [46,47,48]. Weighted analysis of DTW-100 scores for LFP and EMG in the frequency domain before and after stimulation revealed that the magnitude of change induced by LIPUS was more significant in the frequency domain than in the time domain. This further supports the conclusion that LIPUS exerts a more pronounced effect on enhancing neural excitation regarding frequency characteristics and energy levels.

In summary, the enhancing effect of LIPUS on DTW-100 scores was validated from both time- and frequency-domain perspectives and through the analysis of both LFP and EMG signals. Moreover, the neuroprotective effect of LIPUS was quantitatively evaluated from the standpoint of functional cortico-muscular coupling (FCMC).

Due to the limited sample size in this study, only an improved percentile-based DTW scoring algorithm was employed to analyze the time- and frequency-domain performance of LFP and EMG signals in mice under LIPUS intervention. Nonetheless, this approach establishes a foundational framework for developing real-time evaluation and assessment systems in future LIPUS experimental platforms and clinical rehabilitation instruments. However, the small sample size remains a limitation, and advanced approaches such as deep learning have not yet been incorporated—an avenue that will be explored in future research.

In this study, we utilized the surgically induced BCAO model in C57BL/6 mice, a well-established and widely used model for ischemic stroke [49]. However, given that not all clinical stroke cases involve global cerebral ischemia, future studies may benefit from employing localized ischemia models—such as distal middle cerebral artery occlusion (dMCAO) or middle cerebral artery occlusion (MCAO)—to better reflect clinical heterogeneity and enhance translational relevance.

As the evaluation and assessment system proposed in this study is intended for future integration into real-time LIPUS systems capable of simultaneous acquisition of modulation and physiological signals, further optimization and in-depth investigation of the underlying algorithms are required. Additionally, incorporating control group-specific algorithms will be essential to expand the range of assessment strategies available for future neuromuscular monitoring and real-time evaluation platforms.

This evaluation system may be broadly applied to the quantitative assessment of a wide range of neuromuscular disorders during LIPUS-based treatment and rehabilitation, thereby providing a scientific foundation for clinical application. This approach is expected to facilitate a paradigm shift in neurorehabilitation—from experience-driven to data-driven practice—and to establish a stronger research foundation for the clinical translation and broader adoption of LIPUS.

## 5. Conclusions

This study validates the enhancing effect of LIPUS on DTW-100 scores from time-and frequency-domain analyses of LFP and EMG signals, and quantitatively evaluates its neuroprotective effect from the perspective of functional cortico-muscular coupling. An improved DTW scoring algorithm was used, but it lays a foundation for future real-time evaluation systems. Future research should explore incorporating deep learning and using more clinically-relevant ischemia models. The proposed evaluation system needs further algorithm optimization and addition of control group-specific algorithms for broader neuromuscular monitoring and real-time evaluation. This system can be widely applied to quantitatively assess neuromuscular disorders during LIPUS-based treatment, promoting a shift from experience-driven to data-driven neurorehabilitation and strengthening the research foundation for LIPUS clinical translation.

## Data Availability

The data presented in this study are available on request from the corresponding author due to privacy reasons.

## Abbreviations

The following abbreviations are used in this manuscript:

LIPUS	Low-Intensity Pulsed Ultrasound Stimulation
LFP	Local Field Potential
EMG	Electromyography
DTW	Dynamic Time Warping
BCAO	Bilateral Carotid Artery Occlusion
M1	Primary motor cortex
FRE	Frequency
FCMC	Functional Cortico-Muscular Coupling

## Appendix A

**Table A1:** All DTW-100 scores for this study.

**BCAO DTW-100 Scores before LIPUS**	**S1**	**S2**	**S3**	**S4**	**S5**	**S6**	**S7**	**S8**	**S9**
LFP	48	51	56	50	43	50	51	38	46
EMG	55	62	63	60	69	71	62	64	65
**BCAO DTW-100 Scores** **after LIPUS**	**S1**	**S2**	**S3**	**S4**	**S5**	**S6**	**S7**	**S8**	**S9**
LFP	81	82	82	82	78	78	78	77	79
EMG	61	63	60	60	53	54	54	53	52
**DTW-100 Scores**	**S1**	**S2**	**S3**	**S4**	**S5**	**S6**	**S7**	**S8**	**S9**
BCAO	51	57	59	54	56	60	56	51	55
BCAO+LIPUS	71	73	71	71	65	66	66	65	65
**BCAO** **FRE DTW-100 Scores before LIPUS**	**S1**	**S2**	**S3**	**S4**	**S5**	**S6**	**S7**	**S8**	**S9**
LFP	42	28	45	15	26	33	32	67	21
EMG	20	6	24	10	11	16	16	21	30
**BCAO** **FRE DTW-100 Scores** **after LIPUS**	**S1**	**S2**	**S3**	**S4**	**S5**	**S6**	**S7**	**S8**	**S9**
LFP	73	76	75	74	72	74	72	72	75
EMG	63	64	63	65	55	55	54	55	54
**FRE DTW-100 Scores**	**S1**	**S2**	**S3**	**S4**	**S5**	**S6**	**S7**	**S8**	**S9**
BCAO	31	17	35	13	19	25	24	44	26
BCAO+LIPUS	68	70	69	70	64	65	63	64	65

## References

[1] Balch M.H.H., Harris H., Chugh D., Gnyawali S., Rink C., Nimjee S.M., Arnold W.D. (2021). Ischemic Stroke-Induced Polyaxonal Innervation at the Neuromuscular Junction Is Attenuated by Robot-Assisted Mechanical Therapy. Exp. Neurol..

[2] Neumann J.T., Cohan C.H., Dave K.R., Wright C.B., Perez-Pinzon M.A. (2013). Global Cerebral Ischemia: Synaptic and Cognitive Dysfunction. Curr. Drug Targets.

[3] Donnan G.A., Davis S.M., Parsons M.W., Ma H., Dewey H.M., Howells D.W. (2011). How to Make Better Use of Thrombolytic Therapy in Acute Ischemic Stroke. Nat. Rev. Neurol..

[4] Chen Y., Bogosavljevic V., Leys D., Jovanovic D., Beslac-Bumbasirevic L., Lucas C. (2011). Intravenous Thrombolytic Therapy in Patients with Stroke Mimics: Baseline Characteristics and Safety Profile. Eur. J. Neurol..

[5] Marshall R.S. (2015). Progress in Intravenous Thrombolytic Therapy for Acute Stroke. JAMA Neurol..

[6] Meyers P.M., Schumacher H.C., Connolly E.S., Heyer E.J., Gray W.A., Higashida R.T. (2011). Current Status of Endovascular Stroke Treatment. Circulation.

[7] Ding D. (2015). Endovascular Mechanical Thrombectomy for Acute Ischemic Stroke: A New Standard of Care. J. Stroke.

[8] Przybylowski C.J. (2014). Evolution of Endovascular Mechanical Thrombectomy for Acute Ischemic Stroke. World J. Clin. Cases.

[9] Ovbiagele B., Kidwell C.S., Starkman S., Saver J.L. (2003). Neuroprotective Agents for the Treatment of Acute Ischemic Stroke. Curr. Neurol. Neurosci. Rep..

[10] Fisher M. (2011). New Approaches to Neuroprotective Drug Development. Stroke.

[11] Kikuchi K., Uchikado H., Morioka M., Murai Y., Tanaka E. (2012). Clinical Neuroprotective Drugs for Treatment and Prevention of Stroke. Int. J. Mol. Sci..

[12] Jiang X., Savchenko O., Li Y., Qi S., Yang T., Zhang W., Chen J. (2019). A Review of Low-Intensity Pulsed Ultrasound for Therapeutic Applications. IEEE Trans. Bio-Med. Eng..

[13] Chen C.-M., Wu C.-T., Yang T.-H., Liu S.-H., Yang F.-Y. (2018). Preventive Effect of Low Intensity Pulsed Ultrasound against Experimental Cerebral Ischemia/Reperfusion Injury via Apoptosis Reduction and Brain-Derived Neurotrophic Factor Induction. Sci. Rep..

[14] Wu C.-T., Yang T.-H., Chen M.-C., Chung Y.-P., Guan S.-S., Long L.-H., Liu S.-H., Chen C.-M. (2019). Low Intensity Pulsed Ultrasound Prevents Recurrent Ischemic Stroke in a Cerebral Ischemia/Reperfusion Injury Mouse Model via Brain-Derived Neurotrophic Factor Induction. Int. J. Mol. Sci..

[15] Chen J., Zhang X., Zhang C., Wang W., Chen R., Jiao H., Li L., Zhang L., Cui L. (2016). Anti-Inflammation of Natural Components from Medicinal Plants at Low Concentrations in Brain via Inhibiting Neutrophil Infiltration after Stroke. Mediat. Inflamm..

[16] Guo T., Li H., Lv Y., Lu H., Niu J., Sun J., Yang G.-Y., Ren C., Tong S. (2015). Pulsed Transcranial Ultrasound Stimulation Immediately after the Ischemic Brain Injury Is Neuroprotective. IEEE Trans. Bio-Med. Eng..

[17] Su W.-S., Wu C.-H., Chen S.-F., Yang F.-Y. (2017). Low-Intensity Pulsed Ultrasound Improves Behavioral and Histological Outcomes after Experimental Traumatic Brain Injury. Sci. Rep..

[18] Clark D.J., Ting L.H., Zajac F.E., Neptune R.R., Kautz S.A. (2010). Merging of Healthy Motor Modules Predicts Reduced Locomotor Performance and Muscle Coordination Complexity Post-Stroke. J. Neurophysiol..

[19] Arya K.N., Pandian S. (2014). Interlimb Neural Coupling: Implications for Poststroke Hemiparesis. Ann. Phys. Rehabil. Med..

[20] Akbas T., Neptune R.R., Sulzer J. (2019). Neuromusculoskeletal Simulation Reveals Abnormal Rectus Femoris-Gluteus Medius Coupling in Post-Stroke Gait. Front. Neurol..

[21] Levin M.F., Kleim J.A., Wolf S.L. (2009). What Do Motor “Recovery” and “Compensation” Mean in Patients Following Stroke?. Neurorehabil. Neural Repair.

[22] Cai S., Lu Z., Chen B., Guo L., Qing Z., Yao L. (2022). Dynamic Gesture Recognition of A-Mode Ultrasonic Based on the DTW Algorithm. IEEE Sens. J..

[23] Permanasari Y., Harahap E.H., Ali E.P. (2019). Speech Recognition Using Dynamic Time Warping (DTW). J. Phys. Conf. Ser..

[24] Mohan B.J., Babu N.R. (2014). Speech Recognition Using MFCC and DTW. Proceedings of the 2014 International Conference on Advances in Electrical Engineering (ICAEE).

[25] Zhi-Qiang H., Jia-Qi Z., Xin W., Zi-Wei L., Yong L. (2019). Improved Algorithm of DTW in Speech Recognition. IOP Conf. Ser.Mater. Sci. Eng..

[26] Yadav M., Alam M.A. (2018). Dynamic Time Warping (Dtw) Algorithm in Speech: A Review. Int. J. Res. Electron. Comput. Eng..

[27] Yuan Y., Chen Y.-P.P., Ni S., Xu A.G., Tang L., Vingron M., Somel M., Khaitovich P. (2011). Development and Application of a Modified Dynamic Time Warping Algorithm (DTW-S) to Analyses of Primate Brain Expression Time Series. BMC Bioinf..

[28] Cavill R., Kleinjans J., Briedé J.-J. (2013). DTW4Omics: Comparing Patterns in Biological Time Series. PLoS ONE.

[29] Furlanello C., Merler S., Jurman G. (2006). Combining Feature Selection and DTW for Time-Varying Functional Genomics. IEEE Trans. Signal Process..

[30] Han T., Peng Q., Zhu Z., Shen Y., Huang H., Abid N.N. (2020). A Pattern Representation of Stock Time Series Based on DTW. Phys. A.

[31] Han M. (2024). Systematic Financial Risk Detection Based on DTW Dynamic Algorithm and Sensor Network. Meas. Sens..

[32] Tsinaslanidis P.E., Alexandridis A., Zapranis A., Livanis E. Dynamic Time Warping as a Similarity Measure: Applications in Finance. Proceedings of the Hellenic Finance and Accounting Association.

[33] Zhang Z., Tang P., Duan R. (2015). Dynamic Time Warping under Pointwise Shape Context. Inf. Sci..

[34] Li Y., Zhang X., Gong Y., Cheng Y., Gao X., Chen X. (2017). Motor Function Evaluation of Hemiplegic Upper-Extremities Using Data Fusion from Wearable Inertial and Surface EMG Sensors. Sensors.

[35] Hua X., Li J., Wang T., Wang J., Pi S., Li H., Xi X. (2023). Evaluation of Movement Functional Rehabilitation after Stroke: A Study via Graph Theory and Corticomuscular Coupling as Potential Biomarker. Math. Biosci. Eng..

[36] Strojnik V., Komi P.V. (1998). Neuromuscular Fatigue after Maximal Stretch-Shortening Cycle Exercise. J. Appl. Physiol..

[37] Jin Z., Chen X., Du Z., Yuan Y., Li X., Xie P. (2024). Multi-Scale Coupling between LFP and EMG in Mice by Low-Intensity Pulsed Ultrasound Stimulation with Different Number of Tone-Burst. IEEE Trans. Neural Syst. Rehabil. Eng..

[38] Kim E., Kum J., Lee S.H., Kim H. (2022). Development of a Wireless Ultrasonic Brain Stimulation System for Concurrent Bilateral Neuromodulation in Freely Moving Rodents. Front. Neurosci..

[39] Li X.-H., Guo D., Chen L.-Q., Chang Z.-H., Shi J.-X., Hu N., Chen C., Zhang X.-W., Bao S.-Q., Chen M.-M. (2024). Low-Intensity Ultrasound Ameliorates Brain Organoid Integration and Rescues Microcephaly Deficits. Brain.

[40] Song D., Chen X., Zhou N., Yuan Y., Geng S., Zhang C., Zhao Z., Wang X., Bao X., Lan X. (2023). Low-Intensity Pulsed Ultrasound Triggers a Beneficial Neuromodulation in Dementia Mice with Chronic Cerebral Hypoperfusion via Activation of Hippocampal Fndc5/Irisin Signaling. J. Transl. Med..

[41] Remsik A.B., Williams L., Gjini K., Dodd K., Thoma J., Jacobson T., Walczak M., McMillan M., Rajan S., Young B.M. (2019). Ipsilesional Mu Rhythm Desynchronization and Changes in Motor Behavior Following Post Stroke BCI Intervention for Motor Rehabilitation. Front. Neurosci..

[42] Wang I.-T.J., Allen M., Goffin D., Zhu X., Fairless A.H., Brodkin E.S., Siegel S.J., Marsh E.D., Blendy J.A., Zhou Z. (2012). Loss of CDKL5 Disrupts Kinome Profile and Event-Related Potentials Leading to Autistic-like Phenotypes in Mice. Proc. Natl. Acad. Sci. USA.

[43] Heinemann U., Buchheim K., Gabriel S., Kann O., Kovacs R., Schuchmann S. (2002). Cell Death and Metabolic Activity during Epileptiform Discharges and Status Epilepticus in the Hippocampus. Progress in Brain Research.

[44] Reed E.S. (1989). Neural Regulation of Adaptive Behavior. Ecol. Psychol..

[45] Wolpaw J.R., Kamesar A. (2022). Heksor: The Central Nervous System Substrate of an Adaptive Behaviour. J. Physiol..

[46] Voss P., Thomas M.E., Cisneros-Franco J.M., De Villers-Sidani É. (2017). Dynamic Brains and the Changing Rules of Neuroplasticity: Implications for Learning and Recovery. Front. Psychol..

[47] Cramer S.C., Sur M., Dobkin B.H., O’Brien C., Sanger T.D., Trojanowski J.Q., Rumsey J.M., Hicks R., Cameron J., Chen D. (2011). Harnessing Neuroplasticity for Clinical Applications. Brain.

[48] Bottasso E. (2019). Toward the Existence of a Sympathetic Neuroplasticity Adaptive Mechanism Influencing the Immune Response. A Hypothetical View—Part II. Front. Endocrinol..

[49] Tamiya S., Yoshida Y., Harada S., Nakamoto K., Tokuyama S. (2013). Establishment of a Central Post-Stroke Pain Model Using Global Cerebral Ischaemic Mice. J. Pharm. Pharmacol..

